# Nanoplasmonics in Paper-Based Analytical Devices

**DOI:** 10.3389/fbioe.2019.00069

**Published:** 2019-03-29

**Authors:** Salomón Marquez, Eden Morales-Narváez

**Affiliations:** Biophotonic Nanosensors Laboratory, Centro de Investigaciones en Óptica A. C., León, Mexico

**Keywords:** metal nanoparticles, cellulose, biosensors, plasmonic color, microfluidics

## Abstract

Chemical and biological sensing are crucial tools in science and technology. Plasmonic nanoparticles offer a virtually limitless number of photons for sensing applications, which can be available for visual detection over long periods. Moreover, cellulosic materials, such as paper, represent a versatile building block for implementation of simple, yet valuable, microfluidic analytical devices. This mini review outlines the basic theory of nanoplasmonics and the usability of paper as a nanoplasmonic substrate exploiting its features as a (bio)sensing platform based on different mechanisms depending on localized surface plasmon resonance response. Progress, current trends, challenges and opportunities are also underscored. It is intended for general researchers and technologists who are new to the topic as well as specialist/experts in the field.

## Introduction

The quantitative description of plasmons in gold nanoparticles (Mie, 1908), has portrayed great advances not only in physics and chemistry, but also in applications in different areas ranging from biology (Polavarapu et al., 2014), and medicine (Brigger et al., 2012) to energy (Baffou and Quidant, 2012). The ability to confine several orders of magnitude the electromagnetic field at the interface between a metal nanoscale element and a dielectric environment has been exploited as source of heat, light and hot electrons (Baffou and Quidant, 2014). This phenomenon is known as Localized Surface Plasmon Resonance (LSPR). In this regard, nanoplasmonics involves a powerful spectroscopic technique able to track changes occurring at the surrounding environment with a penetration depth of the order of 100 nm (Read et al., 2013) opening a venue for (bio)sensing applications (Dahlin et al., 2013).

The most common configuration employed in nanoplasmonics is via the colloidal suspensions of noble metal nanoparticles. Several researchers have deeply studied and optimized the synthesis of nanoparticles in suspension with the aim of tuning the LPSR response by means of their size, shape and separation distance (Ghosh and Pal, 2007). However, a powerful spectroscopy technique such as LSPR should not be limited to the realm of colloidal suspensions, delimiting its capability for producing wearable (bio)sensors and devices. On the other hand, suitable building blocks for implementation of plasmonic devices have been barely developed (Naik et al., 2013). In this context, due to their versatility, paper-based building blocks offer excellent opportunity as an alternative configuration.

Paper has gained much interest in biology and chemistry primarily because of its low-cost, easy use and fabrication, optical transparency, and biocompatibility. For example, filter paper (Whatman 1) has been employed for analyte separation and identification, a technique named as “Paper Chromatography” that takes advantage of the hydrophilicity of paper to run samples passively (Block et al., 1955). This feature is due to the cellulosic composition of paper (Moon et al., 2011). By exploiting the aforementioned properties of paper, a given type of devices named microfluidic paper-based analytical devices (μPADs) have been used for (bio)sensing applications (Carrilho et al., 2009). These particular types of devices use plasmonic color (colorimetry) as the basic transduction principle for analyte identification.

Herein, we review several strategies for fabricating nanoplasmonic paper-based (bio)sensor devices from design, fabrication and potential applications. Although other reports have introduced the importance of paper-based devices and nanoplasmonics (Nery and Kubota, 2013; Xia et al., 2016; Park et al., 2018), we emphasize the role that microfluidics plays toward the next generation of Do-It-Yourself μPADs devices for programmed liquid handling, mass transport effects and kinetics of the reaction analyses. Moreover, we offer an overview of the nanoplasmonic sensing principles exploited in these devices including concentration of plasmonic nanoparticles, inter-particle/size modulation, *in-situ* formation and surface enhanced Raman scattering. The first section scopes the fundamentals of nanoplasmonics. Next, we illustrate how paper-based substrates can be fabricated and integrated with flow control mechanisms through microfluidics on paper. Special emphasis is given to 2D and 3D paper configurations along with lateral and vertical flow approaches. The last section highlights the sensing mechanisms underlying metal nanoparticles incorporated into paper substrates, particularly for analytical applications.

## Basics of Nanoplasmonics

**Scheme 1:**
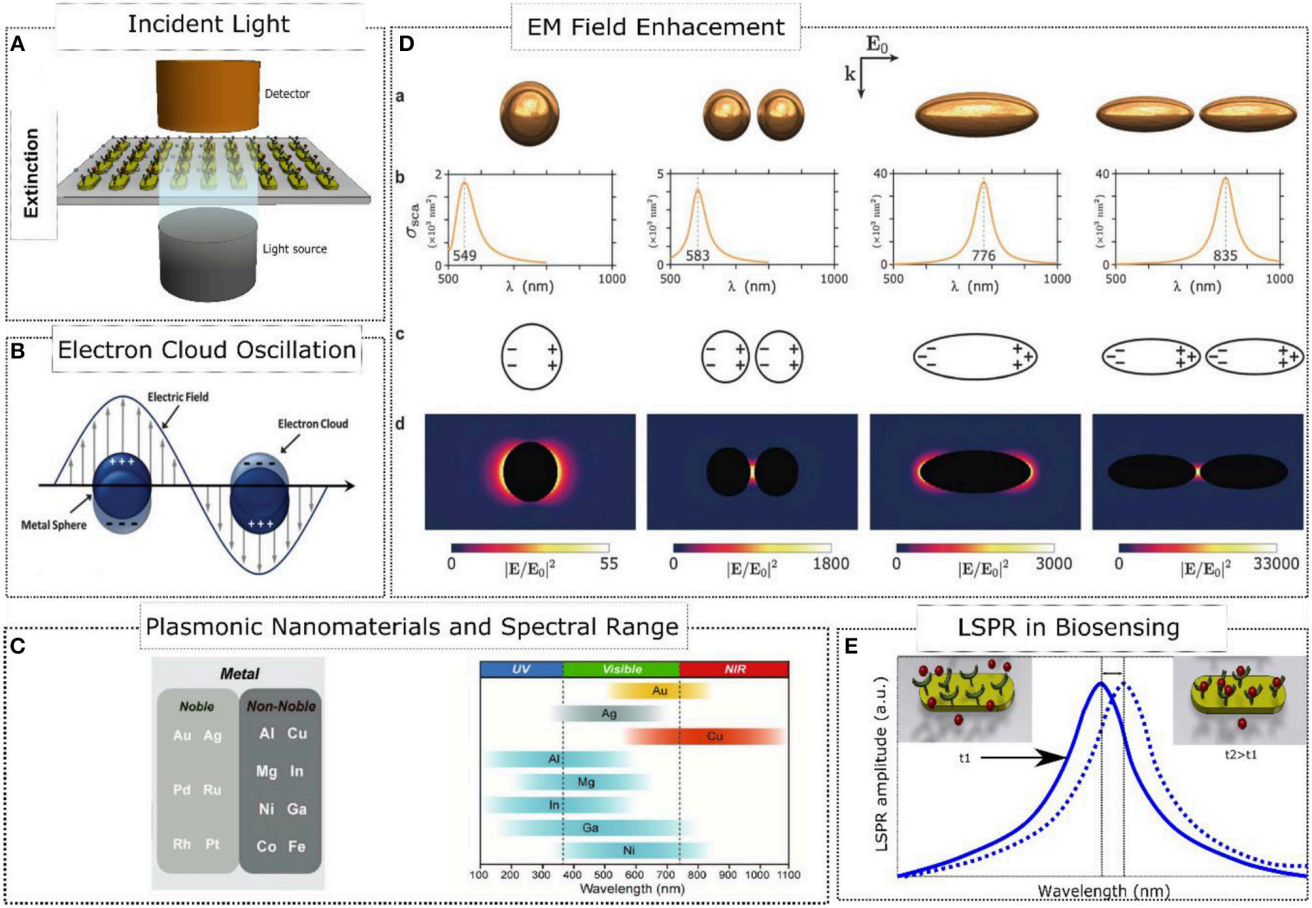
Nanoplasmonics fundamentals. **(A)** Excitation and Measurement of LSPR. **(B)** Oscillation of surface electrons of the metal-dielectric interface upon illumination. Adapted with permission from Motl et al. (2014). Copyright 2014, Royal Society of Chemistry. **(C)** Plasmonic Nanomaterials and their spectral range response. Adapted with permission from Kim et al. (2018). Copyright 2018, WILEY-VCH Verlag GmbH & Co. KGaA, Weinheim. **(D)** Enhancement of the electromagnetic field in several configurations of plasmonic elements. Adapted with permission from Baffou and Quidant (2014). Copyright 2014, Royal Society of Chemistry. **(E)** Localized surface plasmon resonance in biosensing applications.

Nanoplasmonics deals with optical phenomena occurring at a noble metal-dielectric interface of a nanoscale element. Upon illumination, a metal nanoparticle (MNP) can unfold a nanoplasmonic behavior depending on its size, composition, shape, geometry, and dielectric environment. At nanoscale dimensions, an incident electromagnetic wave behaves like a planar front wave and therefore, focusing of light is not required (see Scheme 1A). What happens next is that the cloud of free electrons at the surface of the metal nanoparticle is displaced by the driven action of the electromagnetic wave conforming an electric dipole (see Scheme 1B). This results in an electronic oscillator commonly named as surface plasmon, whose frequency depends on the mass of the electrons and the magnitude of the restoring force (Stockman, 2011). The electromagnetic dipole re-emits light coherently, and part of this light is confined at the metal nanoparticle surface creating hot spots, which are regions that enhance the electromagnetic field (Baffou and Quidant, 2014). Specifically, when the frequency of incident light matches that of the surface plasmon, a localized surface plasmon resonance (LSPR) is exhibited (Stockman, 2011).

Typically, plasmonic nanoparticles are made of noble metals such as Au and Ag. On the one hand, gold nanoparticles are generally reported to be biocompatible (Meola et al., 2018) and silver nanoparticles are even used in commercial products (Cheng et al., 2011); on the other hand, they are relatively costly. However, alternative cost-efficient materials are also being studied in nanoplasmonics (Kim et al., 2018), including other noble metals (Pd, Ru, Rh, Pt) and non-noble metals (Al, Cu, Mg, In, Ni, Ga, Co, Fe), whose biocompatibility is expected to be carefully assessed in new studies. Scheme 1C displays the limitations of these metal nanoparticles in terms of spectral range. As mentioned above, the nanoplasmonic behavior not only depends on the MNP composition but also on its size, shape and inter-particle distance (see Scheme 1D). For example, an isolated oblate exhibits a redshifting of the LSPR response since the restoring force of the electric dipole is affected by the larger separation distance between opposite charges (see Scheme 1D). If otherwise the size of the nanoparticle decreases, a blueshifting is depicted. Another factor that tunes the LSPR response is the dielectric environment. A dielectric material induces a screening of charges at the metal-dielectric interface reducing the excitation of the plasmon. As a consequence, the restoring force of the electric dipole decreases causing redshifting of the LSPR. This can be exploited in biosensing as a mechanism to track changes occurring at the metal-dielectric interface related to the binding of analytes (Lopez et al., 2016) (see Scheme 1E).

The localized enhancement of the optical field exhibited in metal nanostructures has been exploited in a plethora of fields of study such as label-free biosensing (Endo et al., 2006), photothermal therapy (Huang et al., 2007), cancer therapy (Hirsch et al., 2003), near-field optical spectroscopy (Kalkbrenner et al., 2005), and photo-chemical processes (Ueno et al., 2008). Among the most recent developments in nanoplasmonics (Stockman et al., 2018), we find: single molecule Raman probing (Zhang et al., 2013a), plasmon nanoscopy (Bao et al., 2013), plasmonic metal waveguides (Fang and Sun, 2015), thermoplasmonics (Baffou and Quidant, 2012), quantum plasmonics (Moaied et al., 2017), photo-catalysis (Zhang et al., 2013b), ultrafast plasmonics (Piatkowski et al., 2016), plasmonic nanolasers (Ma et al., 2014), and nanoplasmonic (bio)sensors (Lopez et al., 2016). In the following sections, we emphasize how paper-based substrates and nanoplasmonics can be exploited in analytical tasks, which are advantageous in terms of simplicity, low-cost and easy fabrication.

## Fabrication of paper-based devices and their flow control

Microfluidic paper-based analytical devices (μPADs) are single-use innovative analytical platforms capable of handling fluids and analyzing biochemical samples. The incorporation of microfluidics into paper-based devices results advantageous, in comparison to soft polymers (Qin et al., 2010), due to their low-cost and volume consumption, portability and simple fabrication. Paper-based substrates exhibit hydrophilic properties that enable capillary fluid flow (Moon et al., 2011). This is due to the aggregates of micro/nanometer sized cellulose fibers operating as a porous material (pore size between 0.45 and 11 μm). Thus, importantly, no external pumping of fluid is necessary. As a consequence, the flow behavior of a fluid on paper will depend on the composition of the paper type (filter, chromatographic, nitrocellulose paper, or nanopaper), the dimensions and geometry of the outlined fluidic channel, and whether the flow runs laterally or vertically. For example, filter paper (Whatman 1) is adequate not only for sample loading, and transporting but also for path connectivity and also as absorbent pad (Parolo and Merkoçi, 2013). Nitrocellulose paper is suitable for transporting, loading and immobilizing antibodies, and bioconjugated nanoparticles (Lu et al., 2010). Moreover, nanocellullose paper, a particular type of paper produced by *Acetobacter Xylinum* bacteria, exhibits a nanoscale composition of fibers and pores in the range of 10–100 nm (Morales-Narváez et al., 2015). This nanoscale configuration has been of advantage for embedding MNP in nanopaper with the objective of integrating a nanoplasmonic substrate as an alternative to use colloidal suspensions (Golmohammadi et al., 2017).

Concerning the fabrication of a fluidic channel on paper, several techniques have been proposed to outline hydrophobic regions including conventional photolithography (Martinez et al., 2007), hexane-dissolved PDMS (Bruzewicz et al., 2008), plasma treatment (Li et al., 2008), knife plotting (Fenton et al., 2009), wax printing (Carrilho et al., 2009), laser treatment (Chitnis et al., 2011), and inkjet printing (Abe et al., 2008), among others. In this section, we will critically discuss different fabrication methods and their flow control.

### Shaping of 2D μPADs

**Figure 1:**
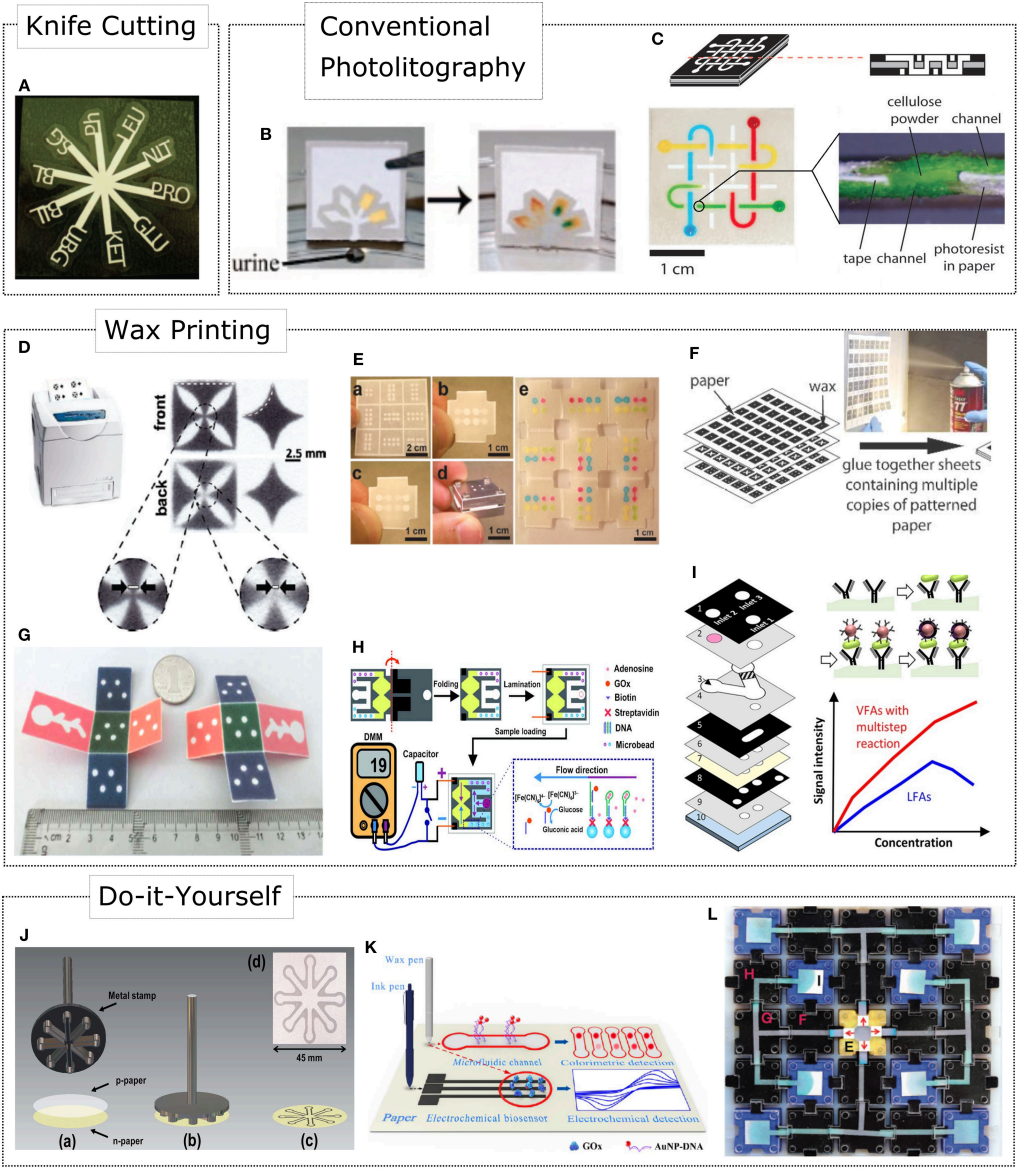
Fabrication methods for integrating fluid handling and control in paper-based devices**. (A)** 2D shaping of cellulose via a knife plotter. Adapted with permission from Fenton et al. (2009). Copyright 2009, American Chemical Society. **(B)** Conventional SU-8 photolithography. Adapted with permission from Martinez et al. (2008a). Copyright 2008, American Chemical Society. **(C)** Stacking of paper layers allows 3D μPADs assembly. Adapted from Martinez et al. (2008b). Copyright 2008, The National Academy of Sciences. **(D)** Two-dimensional shaping of microfluidic devices by wax printing. Adapted with permission from Carrilho et al. (2009). Copyright 2009, American Chemical Society. **(E)** 3D origami paper-based microfluidics (oPADs). Adapted with permission from Liu and Crooks (2011). Copyright 2011, American Chemical Society. **(F)** Spraying a glue to stack multiple 2D designs. Adapted with permission from Lewis et al. (2012). Copyright 2012, The Royal Society of Chemistry. **(G)** 3D origami based CL immunoassay device for multiplexed detection of cancer biomarkers. Adapted with permission from Ge et al. (2012). Copyright 2012, The Royal Society of Chemistry. **(H)** Aptamer-Based oPAD for electrochemical detection of adenosine. Adapted with permission from Liu et al. (2012). Copyright 2012, Wiley-VCH Verlag GmbH & Co. KGaA, Weinheim. **(I)** 3D μPAD enabling vertical flow multistep assays and programmed reagent loading. **(J)** A handheld tool for rapid creation of μPAD by an stamping process. Adapted with permission from Garcia et al. (2014). Copyright 2014, The Royal Society of Chemistry. **(K)** A customized wax pen for creating hydrophobic barriers on filter and nitrocellulose paper. **(L)** A modular paperfluidics platform including prefabricated components to build μPADs. Adapted with permission from Phillips et al. (2018). Copyright 2018, WILEY-VCH Verlag GmbH & Co. KGaA, Weinheim.

Conventional photolithography using SU-8 negative photoresist can pattern high-resolution sub-millimeter fluidic channels on paper substrates (Martinez et al., 2007) (see Figure 1B). However, this is a high cost technique that requires expensive equipment along with organic solvents (Qin et al., 2010). Obviating the need of cleanroom facilities, 2D shaping of cellulose and nitrocellulose paper has allowed rapid prototyping of devices with reduced rates of fabrication errors. This kind of shaping was first carried out via a knife plotter or by using a laser treatment technique (Fenton et al., 2009; Chitnis et al., 2011) (see Figure 1A). The most popular fabrication method of μPADs emerged in 2009: the wax printing technique (Carrilho et al., 2009) (see Figure 1D). This low-cost method allows large-scale and rapid fabrication of devices by following two simple fabrication steps: wax printing of patterns and heat treatment to transfer hydrophobic wax on both sides of paper. Besides, wax printed devices are compatible with most of aqueous solutions except from organic solutions. However, the resolution of the patterned channels is reduced due to wax reflow, and so the minimum resolution achieved for a channel width is around 300 μm outlining hydrophobic barriers of 850 ± 50 μm (Carrilho et al., 2009). Unlike filter paper, nitrocellulose (NC) exhibited attractive advantages such as high protein binding capabilities, controlled fluid flow, and sample purification using the wax printing fabrication method (Lu et al., 2010). All these benefits are due to the micrometer-sized pores of nitrocellulose membranes over 0.45 μm.

### Fabrication of 3D μPADs

The fabrication of 3D μPADs emerged as solution for multiple detection of analytes, where the implementation of more complex microfluidic architectures was necessary. The first 3D μPAD resulted from stacking 2D layers of papers patterned by SU-8 photolithography (Martinez et al., 2008b) (see Figure 1C). Herein, double-sided tape was used to bond the 2D paper layers and cellulose powder to interconnect the fluidic reservoirs for vertical flow of reagents. However, the use of adhesive tape not only required special equipment for shaping reservoirs and channels accurately, but also could contaminate the sample undergoing non-specific absorption. As an alternative solution, a fabrication method of 3D μPADs included a spray to glue and stack multiple layers of 2D designs (Lewis et al., 2012) (see Figure 1F). This resulted in devices with fewer stacked layers facilitating a more rapid vertical flow. The sprayed glue did not affect biocompatibility and hydrophilicity of the devices, but decreased the lateral flow of liquids by 1.3 times.

Similarly, Liu and Crooks (2011) introduced a simple approach to avoid the use of adhesive tape and cellulose powder based on an ancient Japanese tradition to fold paper: the 3D origami paper-based microfluidics (oPADs) (see Figure 1E). The 3D oPADs opened a venue for reversible colorimetric detection with the capability of studying mass transport effects and fluid flow behavior layer-by-layer by simply unfolding each paper layer. Besides colorimetric detection, 3D oPADs have been easily integrated with other transduction mechanisms. For example, multiplexed detection of cancer biomarkers using 3D oPADs was presented by Ge et al. (2012) using a chemiluminescence (CL) immunoassay format (see Figure 1G). Moreover, Liu et al. (2012) introduced an electrochemical transduction scheme with oPADs for detection of adenosine using aptamers as biorecognition probes (see Figure 1H).

Constraining the fluid inside hydrophobic domains is relatively simple in comparison of what microfluidics can contribute to μPADs. 3D configurations of μPADs are also attractive because they can combine both lateral and vertical flow to load samples. For example, Figure 1I shows an approach that implemented a 3D μPAD for programmed reagent loading. This resulted in vertical flow for multistep assays, and a 3.47-fold enhancement for colorimetric detection of C-reactive protein (Park and Park, 2017).

### Do-it-Yourself μPADs

The affordability, biocompatibility and portability of microfluidic paper-based devices have opened the way for creating point-of-care devices in areas with reduced resources. Foremost, if the μPAD is fabricated under a Do-It-Yourself (DYI) format where the user can employ a set of prefabricated microfluidic tools or manufacture the device on his/her own. For example, Lu et al. (2009) developed a wax pen where the design has to be drawn on both sides of the paper. Resolution improvement was achieved by first outlining the pattern using an inkjet printer and then manually drawing the pattern using the wax pen. Although this fabrication technique is simple, it increases reproducibility errors from one user to another. In 2014, Garcia et al. (2014) developed a handheld tool enabling the rapid creation of paper-based microfluidic devices via a stamping process (see Figure 1J). Paraffin is utilized to outline hydrophobic barriers that are transferred onto the paper in <2 min by heating the handheld tool. Recently, an updated version of the wax pen approach was presented by Li et al. (2017) for electrochemical detection of glucose (see Figure 1K). Herein, a customized wax pen and a conductive-ink pen composed by graphite powder and enzyme ink are used to directly write hydrophobic barriers and conductive electrodes, respectively. This versatile tool allows wax flow and penetration on paper directly at 50°C without subsequent heating steps. On the other side, modularity and reconfigurability of paper blocks allowed the study of reaction kinetics for nitrides and isonicotinic acids (Phillips et al., 2018). The Asynchronous Modular Paperfluidics linear instrument-free (Ampli) approach is similar to electronic breadboards where prefabricated components are interchangeable and reusable (see Figure 1L). Programming paper fluidics represents a valuable solution to revolve traditional write-once/read-once architectures of μPADs.

## Nanoplasmonic sensing mechanisms in paper-based devices

The flow control approaches discussed above are often crucial to engineer and implement different sensing mechanisms resulting in advantageous (bio)sensing devices whose results are visually observable. It is worth mentioning that keeping in mind the human vision system and visible spectrum features, the cyan to green transition zone at ~500 nm has been recently reported to be the optimal starting wavelength aiming at visualizing changes in plasmonic color (Chen et al., 2018). In this section, we discuss the main phenomena that underpin the operational principle of paper-based analytical devices exploiting nanoplasmonics.

### Concentration of Plasmonic Nanoparticles

**Figure 2:**
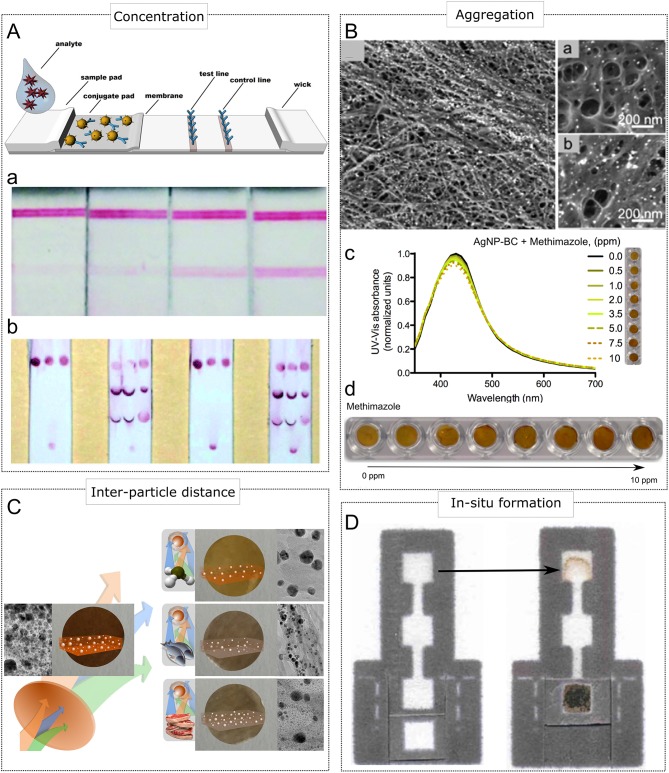
Sensing mechanisms displaying plasmonic color in paper-based devices. **(A)** Concentration of AuNPs in the form of lines **(Aa)** and spots **(Ab)**. **(A)** Adapted with permission from Miočević et al. (2017). Copyright 2017, Frontiers. **(Aa)** Adapted with permission from Rivas et al. (2014). **(Ab)** Adapted with permission from Li and Macdonald (2016). Copyright 2016, Royal Society of Chemistry. **(B)** Aggregation of MNPs. **(Ba)** Nanopaper embedding AgNPs before analyte (methimazole) addition. **(Bb)** Nanopaper embedding aggregated AgNPs upon analyte addition. **(Bc)** Modulation of scattering and absorbance due to AgNPs aggregation and the corresponding changes in plasmonic color. **(Bd)** Adapted with permission from Morales-Narváez et al. (2015). Copyright 2015, American Chemical Society. **(C)** Modulation of plasmonic color due to size changes triggered by etching caused corrosive vapor (AgNPs embedded in nanopaper). Adapted with permission from Heli et al. (2016). Copyright 2016, Royal Society of Chemistry. **(D)** *in situ* generation of AgNPs. Adapted with permission from Hamedpour et al. (2018). Copyright 2018, Springer Nature.

Exclusive areas of cellulosic materials can be functionalized by immobilizing biomaterials onto such areas, for example in the form of lines or spots (Li and Macdonald, 2016). These biomaterials (antibodies and oligonucleotides) or biomimetic materials (molecularly imprinted polymers) are generally employed as biorecognition elements (Morales and Halpern, 2018), thus creating reaction zones interrogating the presence of the analyte upon highly specific and selective interactions occurring between these reaction zones and the analyte previously labeled via plasmonic nanoparticle-decorated biorecognition probes. Consequently, given the flow control provided by paper-based microfluidics and the highly specific interactions facilitated by the involved biorecognition probes, these reaction zones operate as concentration areas of biorecognition events labeled with plasmonic nanoparticles, thus resulting in a visually observable phenomenon reporting the absence/presence of the target molecule. Typically, lateral flow immunoassays reported by gold nanoparticles, such as the pregnancy test, employ this operational principle (Brown, 2009). Figure 2A depicts this clever design. Importantly, lateral flow devices are being employed in several applications, including heavy metal determination and clinically relevant proteins detection at the point of care (Yang et al., 2017; Quesada-González and Merkoçi, 2018). In addition, concentration of plasmonic nanoparticles can also be exploited in μPADs and flow-through devices (Yetisen et al., 2013; Nunes Pauli et al., 2015; López-Marzo and Merkoçi, 2016). Importantly, the sensitivity of this operational principle has been reported to be enhanced by modifying paper architecture (Parolo et al., 2013; Rivas et al., 2014), optimizing nanoparticle size (Zhan et al., 2017) and introducing catalytic amplification techniques (Loynachan et al., 2018).

### Distance and Size Modulation

As highlighted above, scattering and UV-Vis absorbance of plasmonic nanoparticles can be modulated by changing the distance between metal nanoparticles (MNPs). In this context, inter-particle distance can be controlled using specific interactions between the target molecule and MNPs. For example, methimazol, 2-mercaptobenzothiazole, and D-cysteine have been reported to agglomerate silver nanoparticles (AgNPs) embedded in nanopaper (see Figure 2B), thus triggering a change in color from amber to dark amber (Morales-Narváez et al., 2015; Pourreza et al., 2015; Zor, 2018). However, the specificity and selectivity of this sensing principle might be improved and expanded to other kind of analytes by functionalizing MNPs surface via specialized ligands or biorecognition molecules, provided that the interaction of functionalized MNPs undergo agglomeration upon target molecule binding. Hence, specificity and selectivity are quite challenging in this operational principle.

Scattering and UV-Vis absorbance of MNPs can also be strongly modulated by modifying their size. Consequently, etching of MNPs can be judiciously employed as a visually observable sensing mechanism, as well. For example, food spoilage monitoring can be carried out via paper-based devices incorporating MNPs; since food spoilage involves the release of ammonia accompanied by other volatile organic compounds that are able to etch AgNPs (Heli et al., 2016). Interestingly, this phenomenon triggers a change in color in the plasmonic device from amber to grayish color (see Figure 2C). Likewise, UV radiation is able to cause photodegradation of AgNPs, reducing the size of AgNPs incorporated in nanocellulose. This feature can be utilized to monitor safe doses of sun/UV exposure through a change in color in a wearable paper-based device (Barajas-Carmona et al., 2017). As the analyte is responsible for producing an etching onto the utilized MNPs, the specificity and selectivity should be carefully considered and investigated so that the plasmonic device can be successfully developed.

### *In-situ* Formation

Paper-based devices can also take advantage of plasmonic color generated by *in-situ* formation of MNPs, thus facilitating a visually observable interrogation. To this end, a specific analyte can be exploited as a reducing agent of salt precursors containing complex ions with noble metals. For example, uric acid accompanied by NaOH is able to reduce [Ag(NH_3_)_2_]^+^, forming AgNPs and leading to a uric acid sensing platform that can be implemented using paper-based analytical devices (see Figure 2D) (Hamedpour et al., 2018). Specificity and selectivity should also be carefully explored in this kind of approaches.

The enzyme linked immunosorbent assay (ELISA) can be considered a gold standard for protein detection. This immunoassay involves a series of immunoreactions between biomolecules (typically antibodies and antigens) reported by chromogenic agents activated by enzymes labeling such immunoreactions (generally by conjugating detection antibodies with enzymes). Importantly, *in-situ* formation of MNPs can also be enzyme-mediated. For example, growth of gold nanoparticles (AuNPs) can be controlled using catalase (de la Rica and Stevens, 2012), whereas alkaline phosphatase can be employed to generate AgNPs in plasmonic ELISA approaches (Xuan et al., 2016). It is worth mentioning that although ELISA has been transferred to paper-based devices (Cheng et al., 2010), as far as we are concerned, plasmonic ELISA has not been implemented in paper-based platforms. Thus, this represents a challenge, as well as an opportunity in paper-based plasmonic devices.

### Surface Enhanced Raman Scattering

Raman spectroscopy facilitates the determination of (bio)molecules via their highly specific Raman scattering (Haynes et al., 2005), thus leading to a powerful analytical platform which potentially is able to detect analytes at the single molecule level. Importantly, plasmonic nanoparticles are also able to generate a sharp rise in the Raman scattering by means of the local amplification of the electromagnetic field experimented around noble metal nanomaterials, which is caused by the excitation of localized surface plasmon resonances (Gómez and Lazzari, 2014). This phenomenon is known as surface enhanced Raman scattering (SERS). Given their hydrophilicity, flexibility and availability, paper-based SERS substrates (Rodríguez-Sevilla et al., 2018), that is, paper substrates incorporating MNPs designed to enhance Raman spectroscopy response, offer a plethora of advantages in terms of cost, stability, simplicity and fabrication routes. In fact, the literature offers a critical survey related to this topic, covering several cutting-edge applications such as biomedical as well as environmental and food monitoring (Betz et al., 2014; Vicente et al., 2018).

## Perspectives and conclusions

Microfluidics on paper goes beyond confining liquids within hydrophobic domains. As discussed above, there is a special interest focused on the transportation, mixing, separation, and storage of liquids in a controlled and programmed manner. The future of μPADs reported by nanoplasmonics can be approached from two perspectives. The first one is the study of mass transport effects and kinetics of fluids to integrate key control parameters on paper devices such as spacers, delayers, preconcentration, reversible flow zones, and control of the discussed sensing mechanisms. Moreover, the study of reaction time is crucial since these devices are exposed to evaporation of the samples, exposure that can also undergo cross-contamination, undesired nanoparticle agglomeration and the inherent deactivation of reagents. Another factor of great impact is to reduce the number of rinsing steps that are elementary to avoid non-specific absorption in immunosensing approaches. It is notable that these washing steps can markedly reduce the sensitivity of the μPADs due to the effect of dilution and carrying over of analytes in the detection zone that affect the intensity of the resulting nanoplasmonic color. Importantly, a virtually unexplored field of μPADs is the simulation using finite elements that could be of great potential for their preliminary design. The other perspective concerns the manufacturing of μPADs incorporating nanoplasmonics. Currently, the tendency of paper microfluidics is to revolutionize existing traditional architectures in order to create modular devices that can be easily reconfigured to take advantage of their reusability. This concept can be exploited by preferably using a prefabricated set of tools. So, the user can easily implement a μPAD configuration for a particular application obviating the need of a cleanroom facility. In this regard, low-cost and easy to implement fabrication techniques are necessary to open a pathway for manufacturing μPADs reported by nanoplasmonics in a DYI format.

Although the discussed sensing principles are relatively straightforward to implement, their selectivity and specificity should be carefully considered. Moreover, authors working in μPADs generally report analytical parameters such as sensitivity, limit of detection/quantification, accuracy and precision; however, stability across time, and percentage of false negative/positive results are barely reported in the literature, which is critical to achieve real-world applications of nanoplasmonic analytical devices.

All in all, on the one hand, the evolution of μPADs and its related formats offer versatile and flexible platforms to engineer innovative devices. On the other hand, nanoplasmonics brings advantageous optical phenomena than can be exploited as (bio)sensing mechanisms which can be visually observable. Hence, the synergy between nanoplasmonics and paper-based substrates leads to simple, single-use and cost-efficient analytical devices. In fact, these devices are likely to meet the ASSURED criteria (Affordable, Sensitive, Specific, User-friendly, Rapid, Equipment-free, and Delivered to those who require them), which is crucial to further point-of-care and care-at-home devices with potential pre/clinical applications.

## Author Contributions

EM-N conceived the overall manuscript. SM and EM-N contributed to writing, editing, and literature review.

### Conflict of Interest Statement

The authors declare that the research was conducted in the absence of any commercial or financial relationships that could be construed as a potential conflict of interest.
